# A thin ice layer segregates two distinct fungal communities in Antarctic brines from Tarn Flat (Northern Victoria Land)

**DOI:** 10.1038/s41598-018-25079-3

**Published:** 2018-04-26

**Authors:** Luigimaria Borruso, Ciro Sannino, Laura Selbmann, Dario Battistel, Laura Zucconi, Maurizio Azzaro, Benedetta Turchetti, Pietro Buzzini, Mauro Guglielmin

**Affiliations:** 1 Faculty of Science and Technology, Free University of Bozen-Bolzano, Bozen-Bolzano, Italy; 2 Department of Agricultural, Food and Environmental Sciences, University of Perugia, Perugia, Italy; 3 Department of Ecological and Biological Sciences, University of Tuscia, Viterbo, Italy; 4 Italian National Antarctic Museum (MNA), Mycological Section, Genoa, Italy; 5 Department of Environmental Science, Informatics and Statistics, University Ca’ Foscari, Venice, Italy; 6 Institute for the Dynamics of Environmental Processes, IDPA/CNR, Venice, Italy; 7 Institute for Coastal Marine Environment, National Research Council, Messina, Italy; 8 Department of Theoretical and Applied Sciences, Insubria University, Varese, Italy

## Abstract

Brines are hypersaline solutions which have been found within the Antarctic permafrost from the Tarn Flat area (Northern Victoria Land). Here, an investigation on the possible presence and diversity of fungal life within those peculiar ecosystems has been carried out for the first time. Brines samples were collected at 4- and 5-meter depths (TF1 and TF2, respectively), from two brines separated by a thin ice layer. The samples were analyzed via Illumina MiSeq targeting the ITS region specific for both yeasts and filamentous fungi. An unexpected high alpha diversity was found. Beta diversity analysis revealed that the two brines were inhabited by two phylogenetically diverse fungal communities (Unifrac value: 0.56, *p* value < 0.01; Martin’s P-test *p-*value < 0.001) characterized by several specialist taxa. The most abundant fungal genera were *Candida* sp., *Leucosporidium* sp., *Naganishia* sp. and *Sporobolomyces* sp. in TF1, and *Leucosporidium* sp., *Malassezia* sp., *Naganishia* sp. and *Sporobolomyces* sp. in TF2. A few hypotheses on such differentiation have been done: i) the different chemical and physical composition of the brines; ii) the presence *in situ* of a thin layer of ice, acting as a physical barrier; and iii) the diverse geological origin of the brines.

## Introduction

Earth cryosphere includes some of the most extreme environments of the globe that, far to be considered abiotic, harbors a wide diversity of psychrophilic and psychrotolerant microorganisms^1,2^. These highly specialized microorganisms have developed a number of adaptation strategies to overcome the direct and indirect impact of low temperatures on microbial physiology. Besides, the extreme hydro-edaphic conditions in those hostile environments may affect the alpha-diversity of microbial communities^3,4^. In this framework, although Antarctica has been for decades the preferred area for studying the diversity of cold-adapted microorganisms, the ecology and diversity of psychrophilic and psychrotolerant fungi (including yeasts) have been reviewed only in recent years^5–9^. Continental Antarctic brines are hypersaline solutions within permafrost whose high salinity keep them unfrozen several degrees below 0 °C and, depending on the variety of brines, could maintain an aqueous phase even at −50 °C^10^. Their genesis and subsurface pattern of circulation are largely unknown^11–16^. Some of these Antarctic brines occur below ice-sealed lakes^12,13^, below the subglacial lakes^17^ and even underground in ice-free continental areas^15^. Recently, Forte *et al*.^14^ described a new system in which pressurized hypersaline brines were found below an ice-sealed lake, but probably coming from deeper circulation from outside the lake basin.

The recent discovery of brines on Mars surface by the Rover Environmental Monitoring Station on NASA’s Curiosity and the recent hypothesis of near-surface brine mobilization in one of the Jupiter’s satellites (i.e. Europe) has increased the interest in studying Earth analogues environments for possible speculations on the potential extra-terrestrial microbial life^18,19^.

In this context, the characterization of microbiota inhabiting Antarctic brines could also reveal interesting details about their possible geographical and geological origin. Although brines found in Antarctica have stimulated since early 2000 s a large interest as a possible new habitat for both bacterial and algal life^16,20–27^, only a few information are currently available on fungal communities colonizing those peculiar ecosystems^20,24,28,29^. Therefore, a clear picture of mycobiota colonizing Antarctic brines is so far lacking.

In this study, within the framework of the Italian National Research Programme in Antarctica, we have investigated the diversity of fungal communities occurring in hypersaline unfrozen brines found within a perennial frozen lake at Tarn Flat (Northern Victoria Land, Antarctica). In detail, two pockets of liquid brines, separated each other from a few cm layer of ice, were analyzed.

## Results

### Chemical characteristics of the brines

Based on a preliminary direct visual observation, the two brines appeared different each other: the upper one (TF1) was transparent and free of gas while the deeper one (TF2) was yellow coloured and characterized by an intense gas bubbling. Physical and chemical analyses of both TF1 and TF2 are reported in Table 1.

**Table 1:** Chemical parameters of the two brines (TF1 and TF2). The mean (µ) concentration values, standard deviation and *p*-values are reported. Significantly different *p*-value are in bold whereas not significant values are abbreviated with n.s.

Chemical parameters	TF1	TF2	*p*-values
***Anions***			
NO_3_^−^ (g L^−1^)	<0.004	<0.004	
PO_4_^3−^ (g L^−1^)	<0.002	<0.002	
SO_4_^2−^ (g L^−1^)	10.6 (±1.2)	10.9 (±1.6)	n.s.
Total Organic Carbon (mg L^−1^)	82.0 (±0.1)	102.5 (±0.1)	<**0.001**
Total Inorganic Carbon (mg L^−1^)	628.3 (±2.7)	674.8 (±3.2)	<**0.001**
**Trace Elements**			
Li (mg L^−1^)	1.17 (±0.06)	1.77 (±0.9)	n.s.
Mg (g L^−1^)	2.03 (±0.10)	2.80 (±0.15)	<**0.01**
Al (mg L^−1^)	17 ± 2	<5	
K (g L^−1^)	0.54 (±0.03)	0.85 (±0.05)	<**0.01**
Ca (g L^−1^)	0.81 (±0.04)	0.77 (±0.04)	<**0.001**
Ti (mg L^−1^)	263 (±13)	118 (±7)	<**0.01**
V (mg L^−1^)	1.36 (±0.14)	2.89 (±0.30)	<**0.05**
Cr (mg L^−1^)	0.39 (±0.08)	0.23 (±0.05)	<**0.01**
Mn (mg L^−1^)	1.58 (±0.23)	2.05 (±0.32)	n.s
Fe (mg L^−1^)	29.7 (±6.0)	5.7 (±1.1)	<**0.05**
Cu (mg L^−1^)	7.3 (±1.5)	<5	
Zn (mg L^−1^)	17.7 (±3.6)	<5	
Sr (mg L^−1^)	5.7 (±0.8)	7.8 (±1.0)	<**0.01**
I (mg L^−1^)	1.2 (±0.2)	2.0 (±0.3)	<**0.01**
Ba (mg L^−1^)	1.4 (±0.3)	0.8 (±0.2)	<**0.01**
U (µg L^−1^)	7 (±1)	6 (±1)	n.s

The two brines exhibited some differences in terms of chemical characteristics. As already reported in Forte *et al*. TF1 was more saline than TF2 (90 and 75 psu, respectively): based on these data it was possible to estimate a sodium concentration around 20 +/− 3 g L^−1^. Moreover, the two brines significantly differed also in the content of carbonaceous compounds: TOC and TIC where higher in TF2 (Table 1). Trace elements contents also showed that TF1 was significantly enriched in crustal elements such as Al, Ti, Fe, Cu and Zn with respect to TF2 (Table 1). Interestingly, although the two brines showed different salinity values, sulphate content was similar (*p*-value = 0.843).

### Fungal diversity of the brines

**Figure 1:**
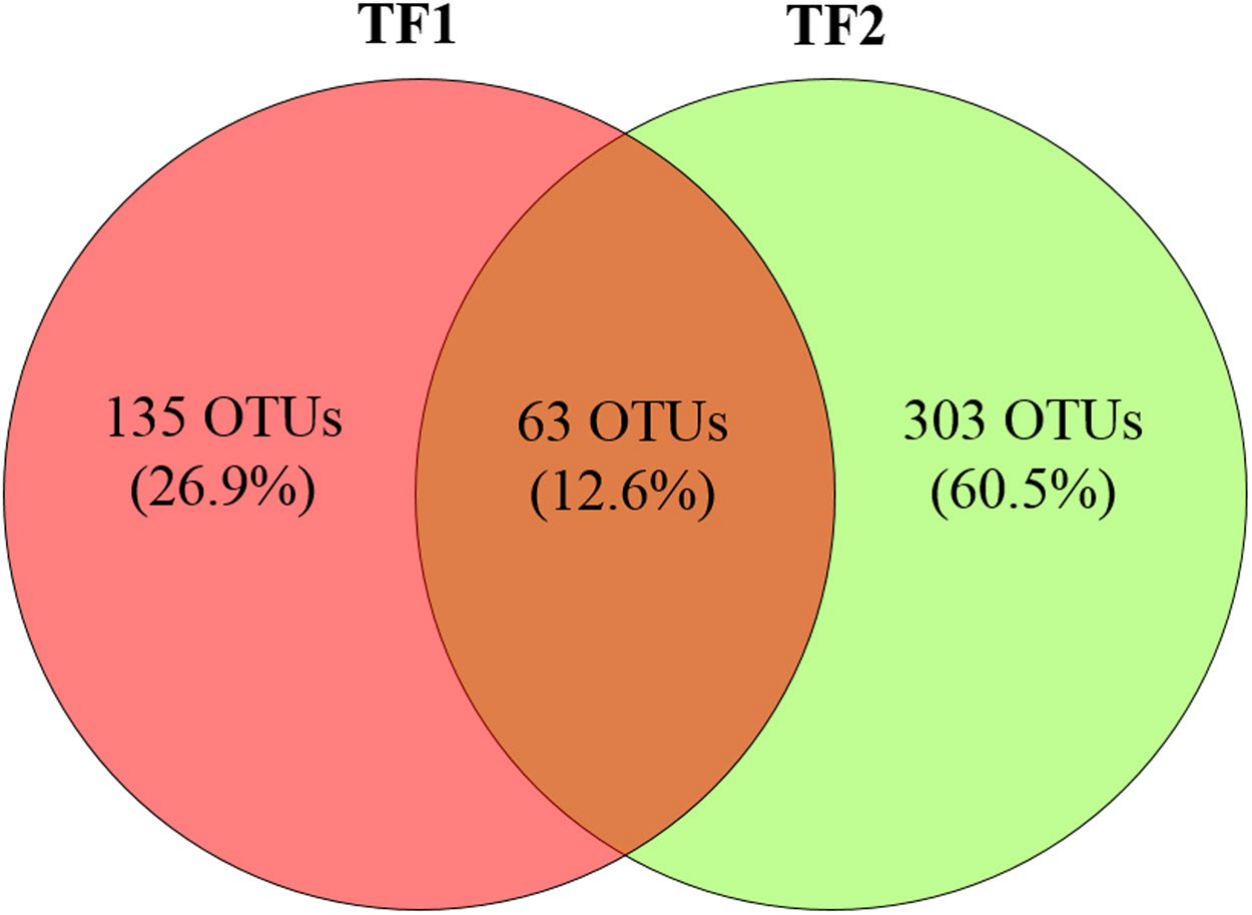
Venn diagram showing the number and percentage of shared fungal OTUs between brine TF1 and TF2. Only OTUs presents in at least two replicates were considered. OTUs were defined by 97% sequence similarity.

Considering all the replicates (n = 3 in TF1 and n = 3 in TF2), after bioinformatics pipeline, a total of 246,928 reads assigned as Fungi clustered in 600 OTUs were found (see Supplementary Tables S1 and S2). They were taxonomically assigned to 3 Phyla, 16 classes, 38 orders, 57 families and 74 genera. Overall, 63 fungal OTUs shared both TF1 and TF2 (12.6% of the total), while 135 and 303 (26.9 and 60.5%) were found exclusively in TF1 or in TF2, respectively (Fig. 1). At the Genus level, only 35 genera out of 74 (47.3%) shared both brines, whereas 8 and 31 genera (10.8 and 41.9%) were found exclusively in TF1 or in TF2, respectively (Table 2).

**Table 2:** Distribution of common and specialist Taxa between TF1 and TF2.

	TF1 specialist Taxa	TF1 and TF2 common Taxa	TF2 specialist Taxa
Phyla	1	2	0
Classes	0	12	4
Orders	2	21	15
Families	4	27	26
Genera	8	35	31

Fungal communities showed a significantly (*p*-value < 0.01) higher richness in TF2 (number of OTUs observed = 337 ± 20) than in TF1 (208 ± 14). Additionally, Shannon index was higher (*p*-value < 0.05) in TF2 (3.00 ± 0.02) than TF1 (2.95 ± 0.01).

**Figure 2:**
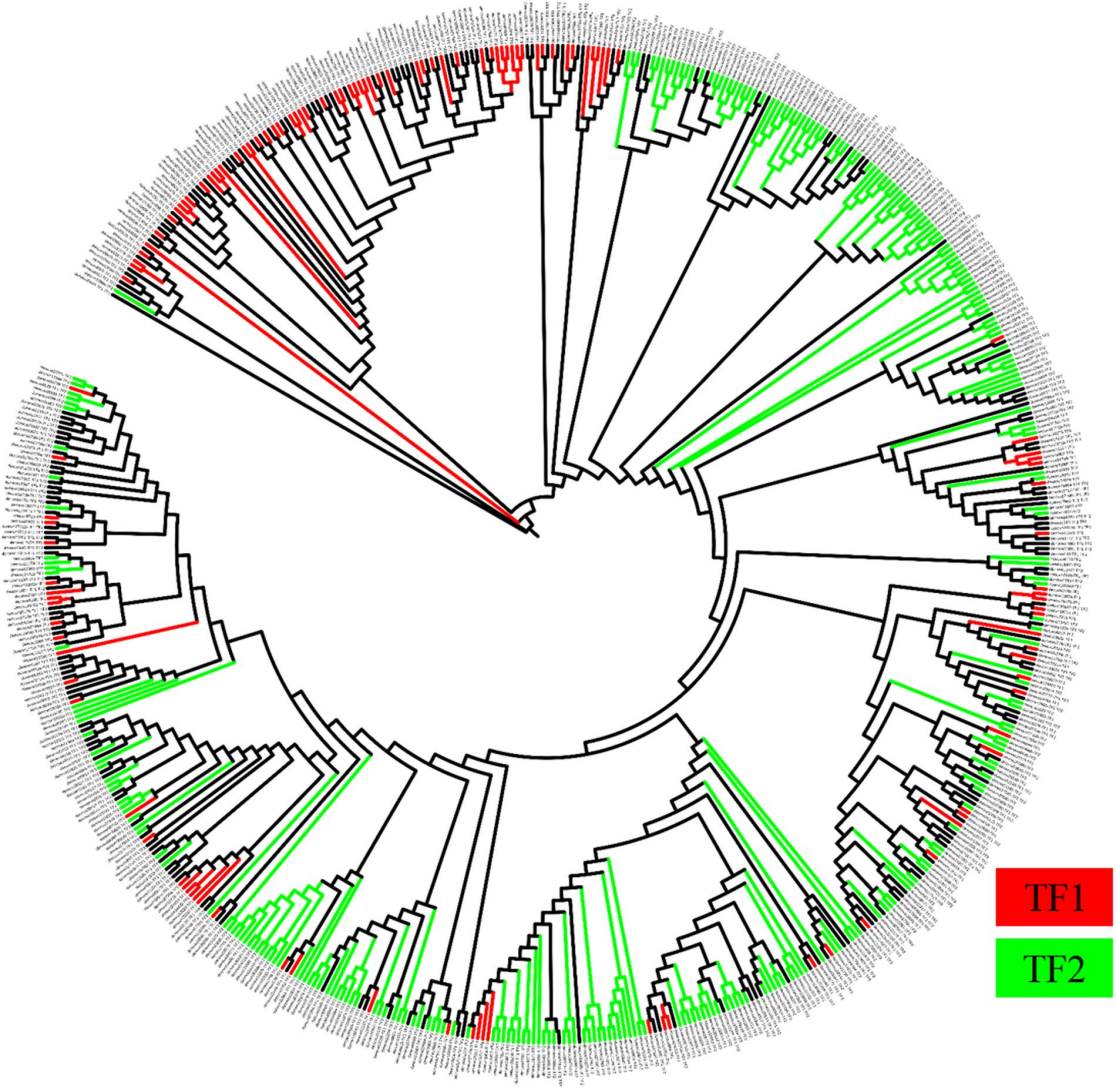
Phylogenetic relationships of the OTUs found in the two brines. Red branches are indicative of fungal OTUs specific of TF1, whereas green OTUs are specific of TF2. Black branches represent fungal OTUs found in both TF1 and TF2.

Martin’s P-test (*p-*value < 0.001) and significant Unifrac test (Unifrac value: 0.56; *p* value: 0.01) revealed that the brines were significantly different. Phylogenetic tree showed that the fungal OTUs found in the two brines were inhabited by two diverse fungal communities characterized by several specialist Taxa (Fig. 2).

### Taxonomic composition of fungal community of the brines

**Figure 3:**
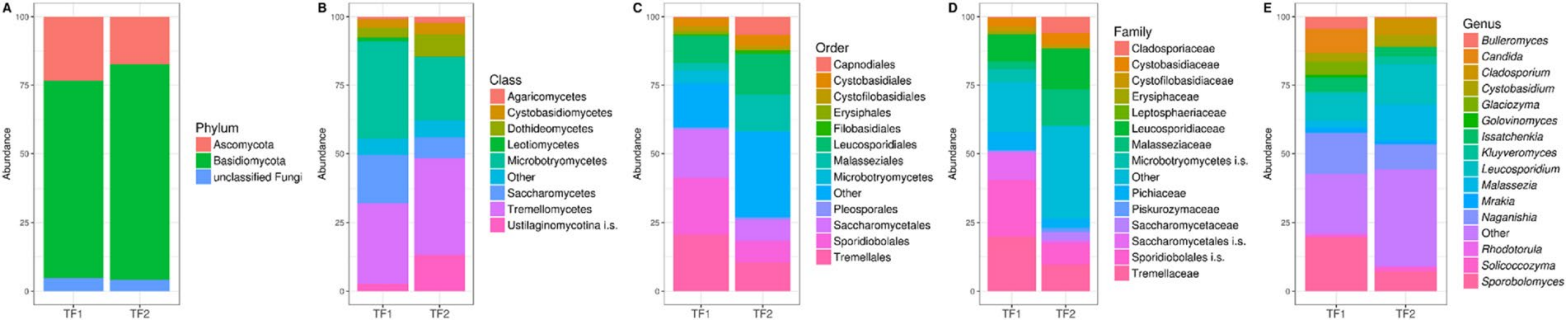
Fungal community composition at Phylum (**A**), Class (**B**), Order (**C**), Family (**D**) and Genus (**E**) level of TF1 and TF2. The data represent the means of three replicates per brine.

Basidiomycota was the dominant phylum in both brines (71.8% in TF1 and 78.6% in TF2), while the OTUs referred as unclassified fungi accounted around 4% in both brines (Fig. 3). However, the taxonomic composition of fungal communities sharing the two brines began to diverge, ranging from classes, orders, families and, finally, to genera. Overall, the number of OTUs referred as “others” (i.e. Taxa found at a level < 1%+ unclassified OTUs in both brines) increased in parallel from class to genera (Fig. 3). Microbotryomycetes, Tremellomycetes and Saccharomycetes were found as dominant (82.3% of total) classes in TF1, whereas Tremellomycetes, Microbotryomycetes, and the subphylum Ustilaginomycotina (referred as *incertae sedis*) accounted for 71.5% of total OTUs in TF2 (Fig. 3). The dominant orders found in TF1, i.e. Leucosporidiales, Tremellales Sporidiobolales, and Saccharomycetales accounted for 68.8% of total OTUs, while Leucosporidiales, Malasseziales, Tremellales and Sporidiobolales were the dominant (46.4%) orders in TF2 (Fig. 3). Sporidiobolales, Saccharomycetales (both referred as *incertae familiae*), Tremellaceae and Leucosporidiaceae were found to be the dominant Taxa at the family level in TF1 (60.3% of total OTUs). On the contrary, Leucosporidiaceae, Malasseziaceae, Tremellaceae and Sporidiobolales (the last referred as *incertae familiae*) dominated in TF2 (45.5%). The family Erysiphaceae was found exclusively in TF1 (about 1%) (Fig. 3). At the genus level, *Candida* sp., *Leucosporidium* sp., *Naganishia* sp. and *Sporobolomyces* sp. accounted for 52.9% of total OTUs found in TF1, whereas *Leucosporidium* sp., *Malassezia* sp., *Naganishia* sp. and *Sporobolomyces* sp. for 44.2% in TF2. The genus *Golovinomyces* (belonging to the family Erysiphaceae) was found exclusively in TF1 (Fig. 3).

## Discussion

Although the present study has limitations due to the low number of brine samples analysed (due to sampling logistic problems), this is the first investigation on the diversity of fungal life within those peculiar Antarctic ecosystems. Furthermore, in McMurdo Dry Valleys brines have been rarely found in some perennially frozen lakes and only in one instance in an underground aquifer^14^.

Geochemical parameters, including total organic and inorganic content, and salinity showed substantial differences in the chemical composition of the two brines. Besides, the relatively high alpha-diversity of fungal communities found in TF1 and TF2 is apparently indicative of fungal communities well adapted to those peculiar ecosystems. Specifically, Shannon and richness diversity were higher in TF2 suggesting a most favourable environment for fungal growth. On the other hands, beta-diversity analysis (Martin’s P-test and Unifrac significance tests) revealed that TF1 and TF2 sequences clustered distinctly and that their overall phylogenetic and taxonomic composition varied significantly due to the presence of diverse fungal lineages in each brine. These data are confirmed by the high number of specialist Taxa detected: over 85% of fungal OTUs were found exclusively in TF1 or in TF2.

The finding of two distinct fungal communities in two brines sampled a few centimeters apart and separated *in situ* only by a thin layer of ice is quite tricky to explain. Maybe, the physical and chemical structures (i.e. nutrient availability, ionic composition and ecological constraints) characterizing the two brines make them different enough to establish such dissimilarity. In addition, the thin ice layer dividing TF1 from TF2 may act as physical barrier, thus probably avoiding the microbial dispersion between the two brines and promoting a microbial divergence due to the local adaptation and/or random genetic drift^30^. Otherwise, the observed diversity of the fungal communities (together with the dissimilar chemical characteristics) found in the two brines could be also related to their possible different origins. Indeed, TF1 can be the results of the infiltration during the summer of surface run off from the lateral partial melting of the lake ice along the shorelines and/or the snow accumulated on the lake and/or around the lake. Therefore, TF1 could have had contacts with the atmosphere and with the surface around the lake. In contrast, TF2 could have been fed by an outside lake basin as suggested by GPR images^14^. This feeding could have enriched TF2 with organic compounds and gases originated from the nutrient rich lake sediments found below TF2 layer in the borehole drilled by Forte *et al*.^14^. On the other hands, TF1 and TF2 shared 12.6% of fungal OTUs. Although the causes of this observed similarity are not easy to explain, we could try to speculate that the thin ice layer segregating the two brines did not persist throughout the time giving the chance of a little exchange of fungal diversity.

Differences of microbial communities colonizing non-Antarctic stratified deep brines have been already investigated. Among them, some hypersaline anoxic basins located in the eastern Mediterranean Sea, namely Urania or L’Atalante^26,31–33^. In those cases, more than 2 meters of brines, characterized by a salinity gradient, were physically separated by different layers, differently from the TF1 and TF2, which were separated only by a few centimeters of ice.

Targeted environmental sequencing of deep-sea sediments from the East Indian Ocean defined 54% yeasts clones related to the former polyphyletic genera *Cryptococcus* and *Rhodotorula*, which have been reported for the majority of deep sub-seafloor samples. On the other hands, phylotypes related to Ascomycota (e.g. *Candida* sp. and *Dipodascus* sp.) were only rarely recovered^34–37^. Lesser abundant yeast taxa (i.e. *Hortaea* sp., *Sporobolomyces* sp. and *Tausonia* sp.) were also found. Fungi represented 17% of total 18 S rRNA gene sequences found in deep-sea super-haline anoxic basins of Bannock and Discovery at the Mediterranean Basin brine and brine/seawater interface, while no fungi were detected in the NaCl-rich Bannock brine and at the Discovery interface. Finally, OTUs related to *Malassezia* spp. and to *Schizosaccharomyces* spp. were found at the Bannock interface^38^.

Interestingly Basidiomycota dominated both TF1 and TF2. This result is in accordance with recent data on the superior ability of basidiomycetes to adapt their physiology to cold conditions^39,40^. However, as above reported, the taxonomic composition of fungal communities sharing the two brines began to diverge, ranging from classes, orders, families, and, finally, to genera. Considering the Taxa detected at genus level, the most abundant genera found in both brines were yeast or yeast-like organisms, suggesting that yeast cell forms can dominate the fungal biodiversity in both brines. This evidence is apparently consistent with previous observations reporting that yeasts are the prevalent form of fungi in the deep-sea habitats characterized by intense salinity^41^. Likewise, Fell^42^ also postulated that the unicellular lifestyle is apparently better adapted to the aqueous environment than fungal hyphae.

In particular, the dominant genera were *Candida*, *Leucosporidium*, *Naganishia* and *Sporobolomyces* in TF1, whereas (with very different relative abundances) *Leucosporidium*, *Malassezia*, *Naganishia* and *Sporobolomyces* in TF2. All above genera include either psychrophilic or psychrotolerant yeasts found in worldwide cold habitats, including Antarctica^9^. Besides, species belonging to the genera *Candida*, *Naganishia* and *Sporobolomyces* were also found in different worldwide saline environments^43^.

The family Erysiphaceae (found exclusively in TF1 with the genus *Golovinomyces*) includes a number of genera of obligate parasites causing powdery mildews on leaves and fruits of higher plants. This genus includes 63 biotrophic, obligate plant pathogenic species^44^ with a host range mostly restricted to herbaceous plants, including up to 2283 species from 58 families^45,46^.

Due to its peculiar ecology, this recovery in Antarctic brines is quite surprising, since angiosperms are totally absent in Continental Antarctica. Besides, fungal spores are easy to disperse even over very long distances and Antarctic environments continuously receive microbial propagules from outside the region^47^. As a result, the microflora found in Antarctic snow, ice, and permafrost may show high frequency of apparently cosmopolitan species^48^, including the ones which ecology is incompatible with Antarctic environments. Therefore, the complex balance between evolution, extinction and colonization may greatly influence the picture of Antarctic microbial diversity.

Fungal OTUs found in TF1 and TF2 represent fungal cells that have most probably remained entrapped in brine for at least several hundred years with temperatures down to −30 °C. The relative low abundance of unclassified OTUs could suggest that communities found in TF1 and TF2, although different, may be not so divergent from the fungal taxonomic structure so far described. This could be due to the transportation of these fungi through the saline brines flows from other sites. Alternatively, they may be resident species but segregated from the global gene pool over a not evolutionary significantly time-scale, as hypothesized for fungi isolated from permafrost samples in the McMurdo Dry Valleys^49^.

As above reported, interest in brines collected in extreme and cold environments has increased after recent observations of recurrent slope lineae (RSL) on Mars as a possible result of the flow of a liquid, likely a saline brine^50–52^. This finding gave special emphasis to the possibility that brines could constitute a terrestrial analogue for studying the possibility of microbial life in sub-surface extraterrestrial (i.e. Martian) habitats^53^. In this context, a few studies^54,55^ described the existence of a possible microbial ecosystem in a deep sub-permafrost talik (890–1,130 m of depth). This deep environment could be considered a terrestrial analog for the Martian subsurface where water could be liquid at kilometers depth beneath the Martian permafrost, thus allowing some speculations on the possible presence of chemoautolithotrophic microbial communities^56^. Of course, thinking that the evolution of life on another planet could have followed exactly the same path as it did on Earth is quite unlikely. However, if TF2 can be considered a brine fed by an open talik system, the possibility to study these environments as possible analogues for speculating on the possible presence of microbial life in sub-permafrost Martian talik could be taken into consideration.

## Materials and Methods

### Study area

**Figure 4:**
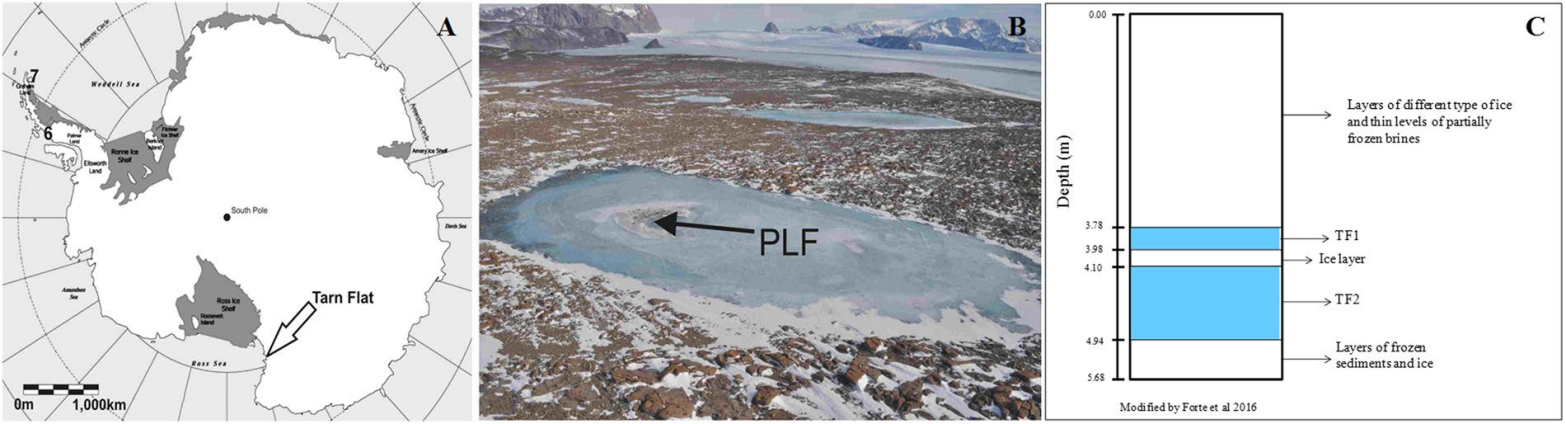
Location of the study area. (**A**) Location of Tarn Flat area within Antarctic continent; (**B**) view of the Tarn Flat area and of the analysed lake in which is clearly visible the PLF indicated by the black arrow where the brines were found; (**C**) Schematic stratigraphy of the borehole (from Forte *et al*., modified)^14^. The light blue levels are the zones where brines (TF1 and TF2) where sampled.

Tarn Flat area (75°4′S 162°30′E) is an ice-free area (ca. 100 km^2^) in the north to the McMurdo Dry Valley in Victoria Land (Antarctica; Fig. 4A). It exhibits a cold and dry climate with a Mean Annual Air Temperature (MAAT) of approximately −14 °C. Precipitations (always in the form of snow) range between 100 and 200 mm/yr^57,58^. During summers, the temperature can reach (or even exceed) +4 °C just for a few days, while the mean seasonal temperature is less than −2 °C^59^.

#### Sample collection

In the studied lake (280 m long and 100 m wide, maximum depth around 6 m) the brines were located only within a deep trough in correspondence of which a pingo like feature (PLF) occurs (Fig. 4B^14^). PLF is a frost mound that intruded the lake ice surface reaching a maximum height of 45 cm and extending within an area of approximately of 500 m^2^. Below that mound, brines have been preliminarily identified using GPR data; they were then reached and sampled through a 51 mm diameter borehole that was drilled in the center of the frost mound using a semi-portable core auger^14^. In particular, the first pocket of liquid brine (TF1) was found between 3.78 m, and 3.98 m (Fig. 4C). TF1 was separated by the second pocket of liquid brine (TF2) (0.84 m thick) by only a 12 cm layer of ice (containing some organic material inclusions). Below TF2 additional frozen sediments rich in organic content occurred between 4.94 m and the bottom of the borehole (5.68 m, Fig. 4C). Both brines were collected in sterile Pyrex bottles using a peristaltic pump and sterile tubing. Additional details of brine sampling are given in Forte *et al*.^14^. After collections, the brines were stored at −20 °C in the dark at Mario Zucchelli Station (MZS) prior to their delivery to laboratories for chemical and microbiological analyses.

#### Chemical analyses

Since salinity and pH in both TF1 and TF2 were already determined and reported in a previous study^14^, in this paper the analysis was mainly focused on the determination of chemical parameters potentially impacting on the heterotrophic metabolism of fungal communities under investigation. In particular, the determination of the organic carbon (TOC) and inorganic carbon (TIC, as carbonate/bicarbonate), nitrate, phosphate, sulphate, and trace elements (TE) such as Li, Mg, Al, K, Ca, Ti, V, Cr, Mn, Fe, Cu, Zn, Sr, I, Ba, U was carried out.

All liquid brine samples were filtered using a PTFE membrane (pore size 0.45 µm) before analyses. Total organic and inorganic carbon contents in the water phase were determined in triplicates using a Shimadzu 5050 A TOC analyzer, following the methodology reported by the manufacturer. The anions (NO_3_^−^, PO_4_^3−^, SO_4_^2−^) were analyzed using ion chromatography (Metrohm 761 Compact IC Chromatography) equipped with a Metrosep A Supp4-250 column. Melted samples were diluted 1:100 before analysis using ultrapure water (ELGA LabWater, Marlow, UK). Three aliquots (n = 3) of each sample were analyzed. For the determination of trace elements (TE), melted samples were diluted 1:10000 (using Ultrapure Water, ELGA), acidified to pH = 1.00 using HNO_3_ (Romil, Cambridge, UK) and analyzed by Inductively Coupled Plasma Sector Field Mass Spectrometry (ICP-SFMS, Finnigan TM ELEMENT2, Thermo Fisher Scientific Inc. Bremen, Germany). Quantification of all chemicals was performed using external calibration curves and the limits of detection were determined from the average values of the procedural blanks (n = 3) plus three standards. Three aliquots of each sample were analyzed (n = 3).

#### DNA extraction

Total DNA from brines samples was aseptically extracted using Power Water DNA Isolation Kit (Qiagen, Germany) following the operating instructions. Prior to DNA extraction, the brines were thawed at 4 °C and aseptically filtered in order to collect the biomass present in each brine on sterile cellulose acetate filter (cutoff = pore size 0.2 µm Sartorius Stedim, Biotech, Germany). The quality and quantity of DNA extracted was determined by using QuBit 2.0 Fluorometer Assay (Life Technologies Corporation) and by NanoDrop 2000 c spectrophotometer (Thermo Fisher Scientific, Waltham, MA, USA).

Three replicates for each brine were run on Illumina MiSeq.

#### Fungal ITS genes data analysis

Fungal internal transcribed spacer region 2 (ITS2) was amplified using IlluAdp_ITS31_NeXTf 5′-CATCGATGAAGAACGCAG-3′ and IlluAdp_ITS4_NeXTr5′-TCCTCCGCTTATTGATATGC-3′^60^. The PCR products were sequenced using the Illumina MiSeq platforms, following the standard protocols of the company STAB Vida Lda. (Caparica, Portugal). Raw data derived from fungal ITS genes Illumina run were processed according to the following pipeline. Paired-end reads from each library were paired via Pear^61^. Sequences with a quality score threshold lower than 30 and shorter than 150 bp were discarded. Assembled reads were analyzed via Qiime v.1.8 software package^62^. Sequences were checked for chimeras with the Chimera VSEARCH^63^. Next, fungal sequences were taxonomically annotated using UNITE + INSDC dataset June 2017 release^64^. Operational taxonomic units (OTUs) table was generated using demultiplexed sequences at 97% similarity and singletons were removed. All sequences have been submitted to the European Nucleotide Archive (EMBL - EBI) under accession number PRJEB23181.

#### Statistical analysis and estimation of fungal alpha and beta diversity

R software was used to perform the statistical analysis^65^. Alpha diversity (richness and Shannon) was estimated using multiple functions of the library vegan^66^. Alpha diversity indices and chemical parameters of TF1 and TF2 were compared using the t-test at a confidence level of 95% for each variable. The statistical equality of variances was firstly evaluated using the F-test. Where the F-test indicates the non-equality of variances (i.e. in the case of lithium and iron), the mean values (µTF) were compared using the Welch’s t-test at a confidence level of 95%.

Venn diagram was generated using Venny^67^, considering only OTUs present in at least two replicates.

Representative OTUs were aligned via MUSCLE version 3.8.31^68,69^ using default parameters. A phylogenetic tree was generated within QIIME using FastTree version 2.1.4^70^ and visualized via Interactive Tree of Life (iTOL) software^71^. Beta diversity was calculated within QIIME using Martin’s P-test and Unifrac significance tests^62,72,73^.

## Electronic supplementary material

Supplementary Dataset 1
