# Supplementary material for: A thin ice layer segregates two distinct fungal communities in Antarctic brines from Tarn Flat (Northern Victoria Land)
Source: Sci Rep. 2018 Apr 26;8:6582. doi: 10.1038/s41598-018-25079-3 (PMC5919928; doi:10.1038/s41598-018-25079-3)
Supplement: Supplementary file 1 — Supplementary Dataset 1 [file 41598_2018_25079_MOESM1_ESM.doc]

**A thin ice layer segregates two distinct fungal communities in Antarctic brines from Tarn Flat (Northern Victoria Land)**

Luigimaria Borruso, Ciro Sannino, Laura Selbmann, Dario Battistel, Laura Zucconi, Maurizio Azzaro, Benedetta Turchetti, Pietro Buzzini, Mauro Guglielmin

Supplementary Table S1: Number of quality filtered fungal sequences and OTUs in each sample.

| Quality filtered reads and OTUs | | |
| --- | --- | --- |
| Samples | Reads | OTUs |
| TF1a | 28731 | 189 |
| TF1b | 27402 | 213 |
| TF1c | 37892 | 223 |
| TF2a | 48693 | 324 |
| TF2b | 46891 | 322 |
| TF2c | 57319 | 365 |

Supplementary Table S2: Statistic of the quality filtered fungal sequences and OTUs.

| Count/sample summary | | | |
| --- | --- | --- | --- |
| Reads | | OTUs | |
| Total count | 246928 | Total count | 600 |
| Min | 27402 | Min | 189 |
| Max | 57319 | Max | 365 |
| Mean | 41155 | Mean | 273 |
| Std. dev. | 10841 | Std. dev. | 67 |
